# The Ability of Bispectral-Guided Management Compared to Routine Monitoring for Reflecting Awareness Rate in Patients Undergoing Abdominal Surgery

**DOI:** 10.5812/ircmj.13584

**Published:** 2014-09-05

**Authors:** Hamedeh Mozafari, Amir Asadi Fakhr, Iraj Salehi, Abbas Moghimbigi

**Affiliations:** 1 Department of Anesthesiology, School of Paramedicine, Hamadan University of Medical Sciences, Hamadan, IR Iran; 2 Modeling of Non Communicable Diseases Research Center, Department of Epidemiology and Biostatistics, School of Public Health, Hamadan University of Medical Sciences, Hamadan, IR Iran

**Keywords:** Anesthesia, Awareness, Consciousness Monitors

## Abstract

**Background:**

Awareness during general anesthesia in different types of surgery is an important described adverse event. Bispectral (BIS) monitoring is one of the recent techniques proposed to monitor the depth of anesthesia.

**Objectives:**

The present study tested the hypothesis that the awareness rate and changes in hemodynamic parameters within anesthesia would be lower in patients allocated to BIS-guided management than those allocated to routine monitoring.

**Materials and Methods:**

In total, 333 adult patients with the American Society of Anesthesiologists (ASA) physical status I-III, aged between 18 and 65 years scheduled for elective abdominal surgery under general anesthesia were included in this randomized double-blind placebo controlled trial. Patients were randomly allocated to BIS monitoring (n = 163) or routine monitoring (n = 170). BIS values and hemodynamic parameters including systolic blood pressure (SBP), diastolic blood pressure (DBP), heart rate (HR), and SPO_2_ were marked before induction (control value), after intubation and laryngoscopy, at intubation, after incision, and also during the operation every 15 minutes until extubation.

**Results:**

The overall incidence of awareness in the BIS and routine monitoring groups were 5.5% and 4.1%, which was not significantly different. There were no significant differences in hemodynamic indices including SBP, DBP, HR, and SPO_2_ before induction of anesthesia between the two groups. These between-group differences in the studied indices remained insignificant at different time points after anesthesia induction as well as post ICU hospitalization. Furthermore, the trend of changes in hemodynamic parameters was comparable in the two groups.

**Conclusions:**

BIS-guided management may not be superior to routine monitoring protocols to prevent awareness as well as hemodynamic changes during general anesthesia in patients undergoing abdominal surgeries.

## 1. Background

Awareness during general anesthesia in different types of surgery is an important adverse event because of its intraoperative physiological responses such as hypertension and tachycardia as well as postoperative psychological sequels such as sleep disturbances, nightmares, daytime anxiety and patients’ dissatisfaction (1-4). This complication can be appeared as the consequence of small doses of administered anesthetic agents or insufficient anesthetic techniques (5). The incidence of awareness during anesthesia is different between 0.10% and 0.20% (6, 7); however, more than 50% of patients had postanesthetic posttraumatic stress disorder. Besides, about 50% to 54% of patients are afraid to wake up during the operation (8). However, it seems that the incidence of this phenomenon and its complications are exactly dependent on the quality of postoperative interview by specialists. It has been shown that detection of awareness depends on the technique, timing and structure of interview (5). Bispectral (BIS) monitoring is one of the recent techniques proposed to monitor the depth of anesthesia derived from electroencephalography (EEG) and measures sedation, hypnosis and loss of consciousness (9-12). By maintaining this indicator between 40 and 60, which is the recommended value for general anesthesia, a reduction of anesthetic requirement and shorter length of stay in post intensive care unit can be predictable (13). Because of its monitoring efficacy, it is now intended to replace other monitoring systems for classifying the depth of anesthesia. The important characteristic of this indexing system is its ability to titrate used anesthetic agents within general anesthesia allowing anesthetists to adjust the amount of anesthetic agent to the needs of patient (14). This might result in a more rapid emergence from anesthesia as well as reducing the incidence of intraoperative awareness in surgeries.

## 2. Objectives

The present study examined whether the awareness rate and changes in vital signs within anesthesia would be lower in patients allocated to BIS-guided management than those allocated to routine monitoring.

## 3. Materials and Methods

Approval was obtained from the Ethics Committee of Hamadan University of Medical Sciences; besides, written informed consent was received from the participants. In total, 333 adult patients of American Society of Anesthesiologists (ASA) physical status I-III, aged 18 to 65 years scheduled for elective abdominal surgery under general anesthesia were included in this randomized double-blind controlled trial. Exclusion criteria were cardiopulmonary disorders, history of head trauma, cerebrovascular accident, psychotic disorders, dementia, depression, history of drug or substances abuse, or lack of sufficient fluency in Persian language. A standard statistical power analysis was performed to determine the size of the randomized, prospective study necessary to demonstrate that the BIS monitor decreases the risk of intraoperative awareness. We considered about 196 samples for each group. Patients were allocated to BIS monitoring (n = 163) and routine monitoring (n = 170) groups using the permuted block randomization method. Nevertheless, 30 and 26 persons disagreed to participate in the study (BIS monitoring n = 163 and routine monitoring n = 170). After the study, we calculated the power of study as 0.91. None of the patients received premedication drugs. A BIS Sensor (danmeter-CSMl) was applied to the forehead and temporal lobe of each patient and BIS parameters were recorded. Routine monitoring included electrocardiography (ECG), noninvasive arterial blood pressure (Saadat Novin s1800 model) and peripheral oxygen saturation (SpO_2_). Anesthesia was induced by IV bolus sufentanil 0.1-0.2 μg/kg, thiopental 3-5 mg/kg and Atracurium 0.5 mg/kg. Anesthesia was maintained with isoflurane or halothane with N2O BIS was used to determine the depth of anesthesia. In the BIS group, anesthesia was maintained by hemodynamic variables and BIS values (target range: 45-65) (10). BIS values and vital parameters including systolic blood pressure (SBP), diastolic blood pressure (DBP), heart rate (HR) and SPO_2_ were recorded before induction (control value), after intubation and laryngoscopy, at intubation, after incision, and during the operation every 15 minutes until extubation. The level of these parameters was recorded in post ICU and after patients’ awaking at 24 hours and 3-7 days after the operation. Baseline characteristics regarding demographics and educational level were collected by face-to-face interviewing. In addition, information related to the awareness during anesthesia was collected by an especial questionnaire including formalized set of open-ended questions. Appropriate reliability and content validity of this questionnaire were previously assessed in the same population in a study by Malek et al. (15). Results were presented as mean ± standard deviation (SD) for quantitative variables and summarized by absolute frequencies and percentages for categorical variables. Categorical variables were compared using Chi-square test or Fisher's exact test when more than 20% of cells with expected count of less than 5 were observed. Quantitative variables were compared using T-test. For statistical analysis, the statistical software SPSS version 19.0 for windows (SPSS Inc., Chicago, IL, USA) was used. P values equal to or less than 0.05 was considered statistically significant.

## 4. Results

Among 333 study participants, 121 patients (36.3%) were male. The most frequent operation was laparoscopy, followed by cholecystectomy. Regarding educational level, 156 patients (46.8%) were illiterate, 84 (25.2%) had a primary educational level, 57 (17.1%) had a secondary educational level, and 36 (10.8%) had higher degree. There were no significant differences between the group with BIS monitoring and those with routine management regarding gender and education level (Table 1). The overall incidence of awareness in the BIS monitoring and routine monitoring groups were 5.5% and 4.1%, but the difference was insignificant.

**Table 1.** Baseline Characteristics of the Study Population^a^

Characteristics	BIS Monitoring Group (n = 163)	Routine Monitoring Group (n = 170)	P Value
**Gender**			0.390
Male	63 (38.7)	58 (34.1)	
Female	100 (61.3)	112 (65.9)	
**Educational level**			0.710
Illiterate	74 (45.4)	82 (48.2)	
Primary level	45 (27.6)	39 (22.9)	
Secondary level	22 (13.5)	35 (20.6)	
College degree	22 (13.5)	14 (8.3)	
**Age, y**	47.389 (18.869)	48.172 (19.212)	0.707
**Awareness within anesthesia**	9 (5.5)	7 (4.1)	0.568

^a^Abbreviation: BIS, bispectral.

**Figure 1.**
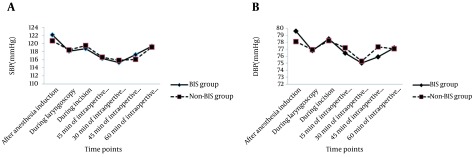
Trend of Changes in Systolic Blood Pressure and Diastolic Blood Pressure A) Repeated measure analysis showed the same changes between the two groups regarding the trend of Systolic BP (F (1.330) = 0.162, P = 0.688); B) F (1.330) = 1.130 P = 0.289.

**Figure 2.**
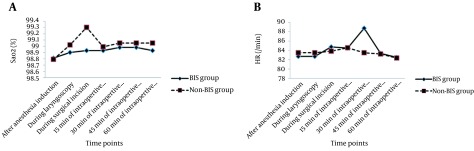
Trend of Changes in SaO_2_ and Heart Rate A) F (1.330) = 3.691, P = 0.055; B) F (1.370) = 0.571, P = 0.450.

There were no significant differences in hemodynamic indices including SBP, DBP, HR, and SPO_2_ before induction of anesthesia between the two groups (Table 2). These between-group differences in the studied indices were remained insignificant at different time points after anesthesia induction as well as within post ICU hospitalization. Furthermore, the trend of changes in hemodynamic parameters was comparable in the two groups (Figures 1 and 2).

**Table 2.** Comparing Changes in Hemodynamic Status in the Study Population^a^

	Systolic BP	Diastolic BP	Arterial O_2_ Sat	Heart Rate
**After anesthesia induction**				
BIS group	122.11 ± 17.49	79.62 ± 12.15	98.81 ± 0.79	82.68 ± 8.77
Non-BIS group	120.65 ± 22.29	78.11 ± 12.12	98.79 ± 1.56	83.45 ± 10.99
P Value	0.510	0.258	0.880	0.486
**During laryngoscopy**				
BIS group	118.09 ± 12.03	76.80 ± 10.07	98.90 ± 0.88	82.68 ± 8.77
Non-BIS group	118.38 ± 16.54	76.92 ± 10.16	99.02 ± 0.65	83.45 ± 10.99
P Value	0.856	0.913	0.173	0.816
**During surgical incision**				
BIS group	118.72 ± 10.87	78.50 ± 9.65	98.93 ± 0.72	84.76 ± 8.55
Non-BIS group	119.51 ± 13.74	78.22 ± 11.99	99.30 ± 0.66	83.79 ± 8.13
P Value	0.565	0.817	0.201	0.291
**First 15-minute of intraoperative period**				
BIS group	116.31 ± 10.93	76.47 ± 9.56	98.93 ± 0.76	84.46 ± 8.56
Non-BIS group	116.63 ± 11.23	77.22 ±8.87	98.99 ± 0.41	84.55 ± 8.47
P Value	0.792	0.459	0.309	0.928
**Second 15-minute of intraoperative period**				
BIS group	115.32 ± 13.29	75.02 ± 9.56	98.98 ± 0.41	88.75 ± 6.28
Non-BIS group	115.83 ± 11.58	75.32 ± 8.87	99.05 ± 0.45	83.50 ± 9.40
P Value	0.709	0.919	0.133	0.230
**Third 15-minute of intraoperative period**				
BIS group	117.25 ± 10.70	75.94 ± 8.51	98.98 ± 0.41	83.37 ± 8.40
Non-BIS group	116.11 ± 12.20	77.33 ± 9.15	99.05 ± 0.45	83.23 ± 8.84
P Value	0.375	0.152	0.267	0.881
**Forth 15-minute of intraoperative period**				
BIS group	119.31 ± 10.40	77.19 ± 8.07	98.93 ± 0.53	82.47 ± 7.05
Non-BIS group	119.09 ± 11.19	77.09 ± 8.43	99.05 ± 0.47	82.36 ± 8.49
P Value	0.856	0.920	0.210	0.886

^a^Abbreviation: BP, blood pressure; Sat, saturation; BIS, bispectral.

## 5. Discussion

In the current study, we tried to determine the desirable monitoring method (BIS monitoring or routine monitoring to prevent re-awareness during general anesthesia. This study of patients undergoing abdominal surgery found no evidence that BIS monitoring reduced awareness within general anesthesia compared to method. Therefore, it seems that this observed insignificant difference is also predictable regarding prevention of awareness-related physiological and psychological complications. In a study by Kertai and his colleagues on patients at high risk for intraoperative awareness, they observed no evidence that BIS monitoring or the avoidance of prolonged periods of BIS values less than 45 improved intermediate-term survival (16). Similarly, Avidan et al. with comparing BIS-based protocol and the protocol based on a measurement of end-tidal anesthetic gas (ETAG) for decreasing anesthesia awareness showed that the level of anesthesia awareness occurred similarly between the groups (3). Besides, numerous studies confirmed the ability of BIS to reduce intermediate outcomes such as hypnotic drug administration, extubation time, postoperative nausea and shortening the recovery room discharge. A recent outcome study using BIS identified an approximately 80% reduction in the incidence of recall after anesthesia (17). Zhang showed that BIS-guided total intravenous anesthesia decreased the risk of awareness compared to routine total intravenous anesthesia (18). Moreover, some authors emphasized BIS monitoring as the clinical standard in general anesthesia (19). The difference between the results of available studies could be due to the association of BIS monitoring outcome with some other variables such as clinical variables and intraoperative factors (20). Totally, we did not reproduce the results of some previous studies reporting a lower incidence of anesthesia awareness with BIS monitoring, and using the BIS protocol might not be associated with reduced administration of anesthetic drugs and gases. Although BIS can provide clinicians with unique information useful to adjust hypnotic drug dosages to individual patient requirements, but it may not be considered as a part of standard practice. In the current study, we also found similar effects of BIS monitoring and routine monitoring on hemodynamic parameters within anesthesia and postoperative periods. Cardiovascular and pulmonary responses to tracheal intubation and other intraoperative techniques are well known and associated with increased catecholamine blood levels (21). In our study, trend of changes in these hemodynamic parameters was not dependent on the type of monitoring technique. In fact, it seems that routine standard anesthesia titration considering hemodynamic parameters is enough for the ASA I-II patients for abdominal surgeries, which was also revealed in previous studies (10, 17).

In conclusion, BIS-guided management is not superior to routine monitoring protocols to prevent awareness as well as hemodynamic changes during general anesthesia in patients undergoing abdominal surgeries.
